# Microbial Population Changes in Decaying *Ascophyllum nodosum* Result in Macroalgal-Polysaccharide-Degrading Bacteria with Potential Applicability in Enzyme-Assisted Extraction Technologies

**DOI:** 10.3390/md17040200

**Published:** 2019-03-29

**Authors:** Maureen W. Ihua, Freddy Guihéneuf, Halimah Mohammed, Lekha M. Margassery, Stephen A. Jackson, Dagmar B. Stengel, David J. Clarke, Alan D. W. Dobson

**Affiliations:** 1 School of Microbiology, University College Cork, Cork, Ireland; w.ihua@umail.ucc.ie (M.W.I.); halimahmoh8@gmail.com (H.M.); lekha513@gmail.com (L.M.M.); sjackson@ucc.ie (S.A.J.); david.clarke@ucc.ie (D.J.C.); 2 Sorbonne Université, CNRS-INSU, Laboratoire d’Océanographie de Villefranche-sur-Mer (LOV), 06230 Villefranche-sur-mer, France; freddy.guiheneuf@invale.com; 3 Botany and Plant Science, School of Natural Sciences, Ryan Institute for Environmental, Marine and Energy Research, National University of Ireland Galway, Galway H91 TK33, Ireland; dagmar.stengel@nuigalway.ie; 4 APC Microbiome Institute, University College Cork, Cork T12 TY20, Ireland; 5 School of Microbiology, Environmental Research Institute, University College Cork, Cork T23 XE10, Ireland

**Keywords:** *Ascophyllum nodosum*, algal cell wall degrading enzymes, enzyme-assisted extraction, ichip device

## Abstract

Seaweeds are of significant interest in the food, pharmaceutical, and agricultural industries as they contain several commercially relevant bioactive compounds. Current extraction methods for macroalgal-derived metabolites are, however, problematic due to the complexity of the algal cell wall which hinders extraction efficiencies. The use of advanced extraction methods, such as enzyme-assisted extraction (EAE), which involve the application of commercial algal cell wall degrading enzymes to hydrolyze the cell wall carbohydrate network, are becoming more popular. *Ascophyllum nodosum* samples were collected from the Irish coast and incubated in artificial seawater for six weeks at three different temperatures (18 °C, 25 °C, and 30 °C) to induce decay. Microbial communities associated with the intact and decaying macroalga were examined using Illumina sequencing and culture-dependent approaches, including the novel ichip device. The bacterial populations associated with the seaweed were observed to change markedly upon decay. Over 800 bacterial isolates cultured from the macroalga were screened for the production of algal cell wall polysaccharidases and a range of species which displayed multiple hydrolytic enzyme activities were identified. Extracts from these enzyme-active bacterial isolates were then used in EAE of phenolics from *Fucus vesiculosus* and were shown to be more efficient than commercial enzyme preparations in their extraction efficiencies.

## 1. Introduction

*Ascophyllum nodosum* (L.) Le Jolis is a brown fucoid which is dominant along the intertidal rocky shores of the North Atlantic [1]. This brown macroalga is of great economic value as an important source of diverse bioactive compounds, many with valuable pharmaceutical, biomedical, and biotechnological potential [2]; which include ascophyllan, laminarin, alginates, and polyphenols [3,4,5]. Extracts from *A. nodosum* have for example been reported to possess potent anticoagulant activity [6], antitumor activity [7], anti-inflammatory activity [8] together with antiviral activity [9], as well as possessing the ability to improve rumen function in animals [10]. Seaweed extracts are also known to help alleviate the consequences of abiotic stress in crops, with extracts from *A. nodosum* being reported to act at the transcriptional level in the model plant *Arabidopsis thaliana* [11] and to also alleviate drought stress in this plant species [12]. Thus, seaweeds and their useful derivatives have become subject to extensive research in recent times.

Macroalgal surfaces are well known to provide a suitable substratum for the attachment of microbial colonizers, including fungi and bacteria, with bacterial densities reaching levels ranging from 10^2^ to 10^7^ cells cm^−2^ [13]. Organic substances secreted by the macroalga act as an important nutritional source for these microorganisms [14,15,16]. Epiphytic bacterial communities are in fact believed to be essential for normal morphological development in the algal host, with these bacteria producing chemicals which help to protect the macroalga from potential harmful secondary colonization by pathogenic microorganisms [17,18]. It is believed that some algal species may contain distinct associated bacterial communities, related to the composition of the algal surfaces and its exudates [19]. Several environmental and non-environmental factors have been shown to influence the composition and abundance of such epibacterial communities associated with seaweeds. In addition to seasonal and temporal variations, the physiological state of the macroalga has also been found to play a significant role in the structure of algal associated microbial communities. Recent studies on *Cladophora*, a filamentous green alga, revealed changes in the diversity and composition of its associated epibacterial communities during decay, to include species involved in nutrient recycling [20].

Marine bacteria are likely to produce cell-wall degrading enzymes as a mechanism to mobilize polymers for nutritional purposes when growing in a nutrient limited state, such as growth on decaying algae. It has been proposed that macroalgal-polysaccharide-degrading (MAPD) bacteria will increase in numbers on weakened or dead macroalgae, thus contributing to recycling of the algal biomass [21]. Several algal polysaccharide-degrading bacteria, which were taxonomically assigned to the *Flavobacteria* and γ-Proteobacteria classes have recently been isolated from the microflora of *A. nodosum* [21]. These bacteria displayed diverse hydrolytic activities and the subsequent functional screening of plurigenomic libraries from these bacteria resulted in the discovery of a range of novel hydrolytic enzymes [22]. Thus, it is clear that the diverse and complex bacterial communities associated with *A. nodosum* represent a potential source of novel hydrolytic enzymes with biotechnological applications that could include enzyme-assisted extraction (EAE) strategies and improvements in the yields of algal components with cosmeceutical, functional food, nutraceutical, and biopharmaceutical applications [23,24].

While seaweeds are rich in useful polysaccharides and other metabolites [25], extraction of such algal components can be problematic due to the complexity and rigidity of the algal cell wall. Brown algal cell walls consist of complex sulfated and branched polysaccharide bound to proteins and ions that hinder extraction efficiencies of algal-derived metabolites [26]. Chemical and mechanical processes are currently used for the extraction of bioactive compounds or fractions from algae; however, problems exist if these compounds are sensitive to extraction techniques which involve heat or the use of solvents [27]. New improved extraction processes including microwave, ultrasound, supercritical fluid, pressurized liquid, and particularly the use of enzyme-assisted extraction (EAE) processes [27], offer ecofriendly, faster, and more efficient alternatives to traditional methods [23,28,29]. Commercial algal cell wall polysaccharidases, such as xylanase, alcalase, viscozyme, neutrase, and agarase, can be used to hydrolyze algal cell wall carbohydrates and eliminate solubility barriers for algal bioactive compounds [27,30]. Enzyme-assisted extraction (EAE) has been successfully employed to produce extracts from the green seaweed *Ulva rigida* (formerly *Ulva armoricana*), which displayed antiviral and antioxidant properties [23]. Carbohydrates and bioactive compounds have also been extracted from the brown algae *Ecklonia radiata* and *Sargassum muticum*, respectively, using carbohydrate hydrolases and proteases in EAE strategies [31].

In this study, our approach was to monitor the overall composition and population dynamics of the microbial communities associated with *A. nodosum* as it decayed over a six-week period by incubating the alga in artificial seawater, using culture independent approaches, targeting the 16S rRNA gene. We also employed culture dependent approaches, including the ichip method [32] to isolate bacteria from the seaweed during the decay process. While bacteria have previously been isolated from macroalgae using traditional cultivation methods [21,33], this study presents the first use of the ichip to cultivate bacteria from decaying seaweed. In this way we hoped to expand the range of bacterial isolates to include previously uncultured microorganisms. Following isolation, bacterial strains were screened for their ability to produce a range of algal cell wall degrading polysaccharidases and were characterized by 16S rRNA DNA sequencing. A range of species from the genus *Bacillus*, together with a number of *Vibrio* species, were isolated, these displayed multiple hydrolytic enzyme activities including hydroxyethyl cellulase, lichenase, and pectinase activities. Extracts from these bacteria were then successfully employed in the EAE of phenolics from the seaweed *Fucus vesiculosus*. 

## 2. Results

### 2.1. MiSeq Sequencing and Data Processing 

The microbial communities of *A. nodosum* samples were analyzed based on decay period (week 0, week 2, week 4, and week 6) and incubating temperature (18 °C, 25 °C, and 30 °C) by Illumina MiSeq sequencing targeting the V3-V4 16S rRNA gene region. A combined total of 8,872,164 raw reads were obtained which, when quality filtered produced, 1,655,910 reads with an average length of 301 bp, and were analyzed using the QIIME version 1.9.1 (http://qiime.org/tutorials/illumina_overview_tutorial.html) workflow [34]. The number of reads obtained after quality filtering and operational taxonomic units (OTUs) together with species richness and diversity indices of the microbial communities are shown in Table 1. With respect to the two diversity indices calculated; Shannon and Chao1, sample 4_30 (week 4; 30 °C) ranked the highest and sample 6_18 (week 6; 18 °C) ranked the lowest in diversity. In addition, it is noteworthy that up to 4% of the OTUs observed in the undecayed *Ascophyllum* sample (T_0_; week 0) and 41% in the decaying samples were unclassified. These unassigned OTUs are likely to represent macroalgal associated bacterial populations that are as yet unknown or not present in the SILVA version 123 database [35] used for taxonomy assignment.

### 2.2. Metagenomic Communities Associated with Intact *Ascophyllum nodosum*

**Figure 1:**
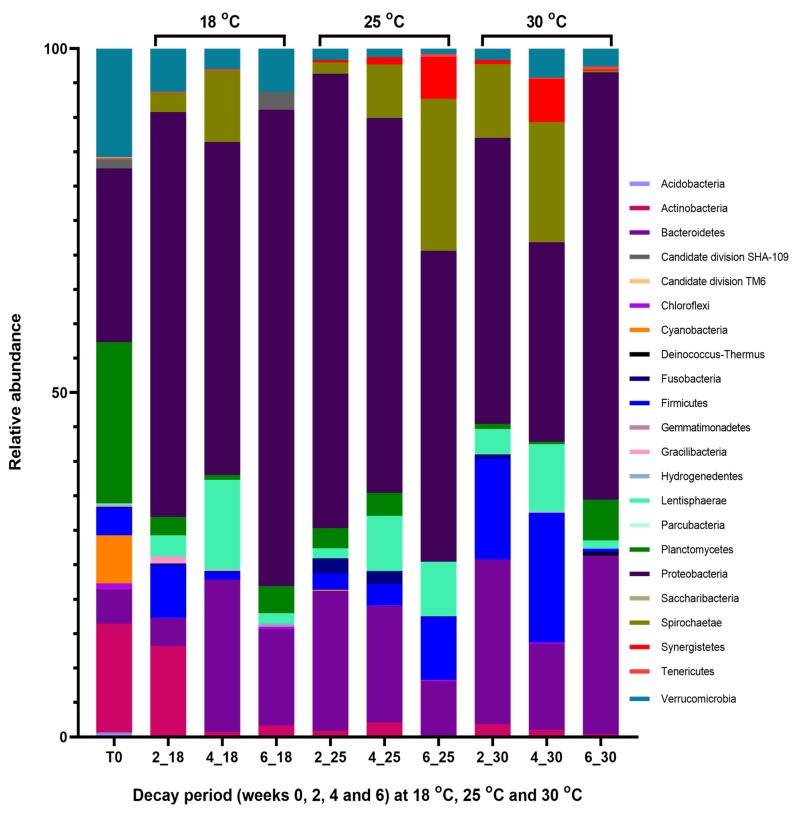
Relative abundances of bacterial phyla associated with intact (T_0_) and decaying *Ascophyllum nodosum* at 2, 4, and 6 weeks of decay at 18 °C (2_18, 4_18, 6_18); 2, 4, and 6 weeks of decay at 25 °C (2_25, 4_25, 6_25), and 2, 4, and 6 weeks of decay at 30 °C (2_30, 4_30, 6_30) obtained from metagenomic 16S rRNA gene sequencing. The relative distribution of phyla in each group is represented as a percentage.

A total of 1467 OTUs were identified among sequence reads derived from the intact undegraded seaweed (T_0_) with approximately 96% of the OTUs been classified into one of 19 different phyla, the major ones being Proteobacteria (24.2%), Planctomycetes (22.5%), Actinobacteria (15.2%), Verrucomicrobia (15.1%), Cyanobacteria (6.7%), Bacteroidetes (4.8%), and Firmicutes (3.8%) (Figure 1; T_0_). The species richness and diversity of the microbial community associated with the macroalga in its intact state as represented by the number of OTUs and number of bacterial phyla identified is also reflected by the Chao1 richness and Shannon diversity index calculated (Table 1).

### 2.3. Metagenomic Communities Associated with Decaying *Ascophyllum nodosum*

#### 2.3.1. Population Changes in the Seaweed Decaying at 18 °C 

Within the first two weeks of the algal decay at 18 °C, the bacterial population associated with the intact seaweed, which was previously characterized by the prevalence of Proteobacteria (24.2%), Planctomycetes (22.5%) and Verrucomicrobia (15.1%), shifted towards a Proteobacteria-led population with a relative abundance of over 40% (Figure 1; 18 °C). This increase in the prevalence of Proteobacteria occurred with a corresponding decline in the relative abundances of some phyla including Planctomycetes (1.8%) and Verrucomicrobia (4.3%), amongst others. However, in the early decay period (at week 2), bacteria belonging to the phylum Spirochaetae which were not identified in the intact seaweed emerged, while other phyla such as Firmicutes, Fusobacteria, Lentisphaerae, and Gracilibacteria were found to be increasingly abundant, relative to their levels prior to the algal decay. The composition of the metagenomic communities associated with the decaying seaweed remained unchanged in the next phase of the algal decay process (at week 4), but changes in the relative abundances of some phyla occurred. At week 4, increase in the presence of bacterial groups classified as Bacteroidetes, Spirochaetae, and Lentisphaerae were more apparent while Proteobacteria levels declined. The bacterial population identified at the end of the decay process (week 6) was less diverse, with 443 OTUs identified and low diversity indices calculated (Table 1). Metagenomic results show that a number of bacterial phyla present in the preceding decay phases were not found at week 6. Some of these bacterial phyla which were not present include Firmicutes, Fusobacteria, Spirochaetae and Gracilibacteria.

#### 2.3.2. Population Changes in the Seaweed Decaying at 25 °C

When compared to the bacterial population associated with fresh *Ascophyllum nodosum* samples, the macroalga allowed to decay at 25 °C experienced a sharp decrease in the relative abundances of bacteria belonging to the phyla Planctomycetes, Verrucomicrobia, Firmicutes and Actinobacteria amongst others in the early decay period. Metagenomic analysis of the 16S rRNA gene sequences revealed a steady shift in the composition and abundance of the microbial communities associated with the decaying seaweed. For example, while the phylum Spirochaetae was not identified prior to decay (T_0_), bacteria classified as this phylum were identified at week 2 (0.9%) and increased in relative abundance throughout the decay period from 3.7% at week 4 to approximately 11% at week 6. Lentisphaerae, which was rarely identified in the undecayed macroalga, was also observed to follow a similar trend, by increasing in relative abundance as *A. nodosum* samples decayed at 25 °C. Levels of this phylum increased from 0.8% at week 2 to 3.8% and 4% at the mid and late phases of the decay period, respectively. Other phyla such as Fusobacteria and Bacteroidetes were observed to decrease consistently in their prevalence from week 2 to week 6 of the decay process (Figure 1; 25 °C). In general, *Ascophyllum nodosum* allowed to decay at 25 °C was dominated by bacteria recruiting to the phylum Proteobacteria. 

#### 2.3.3. Population Changes in the Seaweed Decaying at 30 °C 

At 30 °C, the decaying macroalga was diversely comprised of bacteria belonging to different phyla (Figure 1; 30 °C). The seaweed-associated metagenomic population changed markedly upon decay, relative to the microbial communities found in the intact seaweed (described in Section 2.2) and some of the most notable changes observed occurred in the early decay phase. At week 2, the relative abundance of bacteria belonging to the phylum Actinobacteria was observed to have decreased from approximately 15% (found in week 0) to about 1%. This decrease in relative abundance of Actinobacteria was concurrent with a decline in the prevalence of bacteria belonging to the phyla Planctomycetes (0.4%) and Verrucomicrobia (0.9%) previously present at approximately 22% and 15%, respectively, in the intact seaweed. On the other hand, in the early decay phase, other phyla such as Bacteroidetes, Lentisphaerae, and Firmicutes were observed to increase to more than two-fold in their relative abundances in the decaying seaweed. The microbial population observed at the mid decay phase (week 4) did not differ greatly in its composition from the week 2 derived microbial population. However, the prevalence of Proteobacteria declined (22.4% early phase; 17.1% mid phase) as bacteria identified as Spirochaetae (5.8% early phase; 10.3% mid phase) and Synergistetes (0.3% early phase; 3.7% mid phase) increased in prevalence. Differences in the bacterial phyla present in the decaying seaweed became even more evident at the end of the decay period (week 6) when Proteobacteria regained dominance (43.1%) and the levels of Spirochaetae (0.2%) and Synergistetes (0.1%) declined. 

### 2.4. Cultivable Surface Microbiota Associated with Intact *A. nodosum*

**Figure 2:**
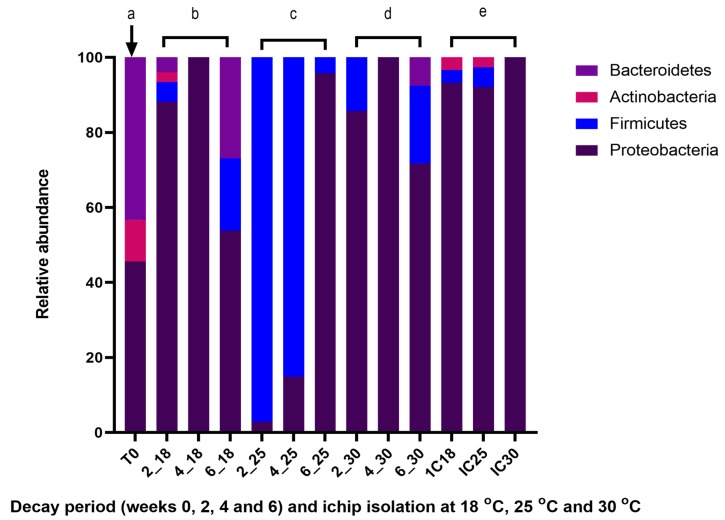
Relative abundances of bacterial phyla associated with the cultivable surface microbiota of (**a**) intact *Ascophyllum nodosum* and decaying *Ascophyllum nodosum* at 2, 4, and 6 weeks of decay at (**b**) 18 °C; 2_18, 4_18, 6_18, (**c**) 25 °C; 2_25, 4_25, 6_25, (**d**) 30 °C; 2_30, 4_30, 6_30 which were obtained by maceration culture isolation method and (**e**) obtained by ichip culture isolation method. 16S rRNA gene sequences were obtained from the bacterial isolates and taxonomic analyses were performed. The relative distribution of phyla in each group is represented as a percentage.

The cultivable epibacterial population of intact and decaying *A. nodosum* samples were assessed using the maceration cultivation method, which involves cutting the seaweed samples into smaller fine pieces. A total of 90 bacteria were isolated and taxonomically identified following 16S rDNA sequence analysis (Table 2) from the intact *Ascophyllum nodosum* sample (T_0_) and were found to consist of bacteria belonging to the phyla Proteobacteria (46%), Bacteroidetes (43%) and Actinobacteria (11%) (Figure 2a). Members of the phylum Proteobacteria were largely dominated by the class γ–Proteobacteria (95%), with α-Proteobacteria and β-Proteobacteria being rarely isolated. In the total isolated bacterial population from the intact seaweed (T_0_), eleven different genera were identified, the most abundantly represented being *Winogradskyella* (41%), *Marinobacter* (37%), *Microbacterium* (6%), and *Micrococcus* (6%) (Figure S1a; Supplementary information). 

### 2.5. Cultivable Surface Microbiota Found on Decaying A. nodosum

The cultivable surface-attached microbiota of the intact seaweed differed greatly from the bacterial populations found on *Ascophyllum* samples allowed to decay at 18 °C, 25 °C, and 30 °C for six weeks (Figure 2). At the phylum level, while Proteobacteria maintained an overall dominance with over 70% relative abundance in the bacterial population associated with both the intact and decaying seaweed, members of the phylum Firmicutes which were not identified in the intact seaweed were present during the algal decay. Bacteria belonging to the phylum Bacteroidetes, which were prevalent in the intact macroalga (43%), represented only 3% of the total cultivable surface microbiota population in the decaying seaweed. 

Similar bacterial phyla were present in the bacterial communities associated with the decaying seaweed in the three incubation flasks (18 °C, 25 °C, and 30 °C). However, distinct differences in the composition of the associated microbial populations found at the different decay periods and temperature are more evident at the genus level (Figure S1; Supplementary information). In the early decay phase, the bacterial population isolated from the macroalga decaying at 18 °C consisted of Proteobacteria (88%), Firmicutes (5%), Bacteroidetes (4%), and Actinobacteria (3%) (Figure 2b) and was diversely comprised of members of the genera; *Paracoccus* (71%), *Celeribacter* (7%), *Psychrobacter* (8%), *Bacillus* (5%), *Formosa* (4%), *Microbacterium* (3%), *Cobetia* (1%), and *Citricella* (1%) (Figure S1b; Supplementary information). Much lower diversity was observed in the bacterial communities isolated at week 2 from the 25 °C and 30 °C incubation flasks, with only members of the phyla Proteobacteria and Firmicutes being identified. 

In the mid decay period (week 4), the genus *Celeribacter* dominated the bacterial population with a relative abundance of 95% and 100% in the 18 °C and 30 °C derived bacterial populations, respectively, while the 25 °C derived population at week 4 was comprised of more genera including *Paenisporosarcina* (51%), *Bacillus* (30%), *Celeribacter* (13%), *Paenibacillus* (2%) *Vibrio* (2%), and *Sporosarcina* (2%) (Figure S1c; Supplementary information). 

Members of the bacterial phyla Proteobacteria (54%), Bacteroidetes (27%), and Firmicutes (19%) were identified in the bacterial population cultured at the end of the decay process (week 6) from the 18 °C incubation flask. These phyla were also present at 72%, 7%, and 21% relative abundances, respectively, in the 30 °C bacterial population (Figure 2d). In week 6, eleven distinct genera were identified at 18 °C, the most abundantly represented being *Paracoccus*, *Algoriphagus*, *Celeribacter*, *Bacillus*, and *Primorskyibacter* and four genera including *Celeribacter*, *Bacillus, Pseudozobellia*, and *Paracoccus* being observed in the 30 °C microbial community. The bacterial community associated with the seaweed decaying at 25 °C in the late decay phase differed from the bacterial communities isolated from the 18 °C and 30 °C microbial populations in this phase, with Proteobacteria (96%) dominating the dataset and three genera; *Celeribacter, Bacillus* and *Roseobacter* being isolated at 25 °C. Phylogenetic trees representing the bacteria cultured from both intact and decaying *Ascophyllum nodosum* samples using the maceration method are shown in Figures S2–S5 (Supplementary information).

### 2.6. ichip Bacterial Isolation Method Applied to Decaying *A. nodosum*

In a bid to further analyze the cultivable bacteria present and potentially expand upon the range of bacterial isolates, the ichip device was also employed on the decaying seaweed samples. At week 4 of the decay period, the ichip device loaded with a cell–agar suspension prepared from the decaying seaweed was inoculated into each flask containing the alga which were decomposing at 18 °C, 25 °C, and 30 °C; with bacteria being recovered from the device following a further 2 weeks of incubation. A total of 224 bacteria (59 isolates from 18 °C, 76 and 89 isolates from 25 °C and 30 °C, respectively; Table 2) were isolated and taxonomically identified using 16S rRNA gene sequences. Taxonomic analysis of the cultivable microbial communities revealed the presence of three representative phyla—Proteobacteria, Actinobacteria, and Firmicutes—across the three different temperature groups. Proteobacteria dominated at the phylum level in the three datasets, comprising 93%, 92%, and 100% in the 18 °C, 25 °C, and 30 °C bacterial culture populations, respectively (Figure 2e). Low relative abundances of Actinobacteria and Firmicutes were observed in the 18 °C and 25 °C populations, with both phyla not being identified in the 30 °C bacterial population. The majority of bacterial isolates cultured from the 18 °C and 25 °C samples, which belong to the phylum Proteobacteria; were further classified as α–Proteobacteria, with only 9–11% recruiting to γ-Proteobacteria. In contrast, γ-Proteobacteria were found to dominate the microbial community isolated from the seaweed incubated at 30 °C, with a relative abundance of 98%. Thirteen distinct genera, including *Celeribacter*, *Paracoccus, Vibrio* and *Marinobacterium* were present in the total bacterial population isolated using the ichip device (Figure S1e; Supplementary information). The genus *Celeribacter* was present across all temperatures, at very high relative abundances in the 18 °C and 25 °C samples (64% and 83%, respectively) but at a much lower relative abundance at 30 °C (2%). In contrast, *Enterobacter*, which dominated at 30 °C, was not found to be present in either of the 18 °C or 25 °C derived microbial populations. The phylogenetic tree representing the bacteria cultured from decaying *Ascophyllum nodosum* samples using the ichip in situ cultivation method is shown in Figure S6 (Supplementary information).

### 2.7. Enzymatic Activities of A. nodosum Cultivable Surface Microbiota 

#### 2.7.1. Intact *Ascophyllum nodosum* Isolated Using the Maceration Method

Over 800 bacterial isolates cultured from intact (T_0_) and decaying *Ascophyllum nodosum* samples using both the maceration and ichip isolation methods (Table 2) were screened for enzyme activity in plate assays containing hydroxyethyl cellulose, pectin, and lichenin as substrates. The cultivable surface microbiota community associated with the seaweed in its intact state was found not to produce any of the algal cell wall degrading enzymes examined under the conditions employed in this study, with none of the bacterial isolates testing positive on any of the plate assays used. 

#### 2.7.2. Decaying *Ascophyllum nodosum* Isolated Using the Maceration Method

The microbial population associated with the decaying seaweed isolated using the maceration method consisted of a total of 51 isolates (approximately 7%) with hydrolytic activity against at least one of the tested substrates (Table S1, Supplementary information). Of these enzyme active bacterial isolates, 65% belonged to the microbial community cultured from the decaying seaweed at week 2, another 10% belonged to the week 4 bacterial population, while 25% were cultured from week 6 and the majority of these MAPD bacteria were found to degrade lichenin (Table S1; Supplementary information). Bacteria belonging to the genus *Bacillus* (10%) represented one of the less abundant genera in the total microbial community associated with the decaying seaweed. However, among the bacteria cultured from the decaying seaweed using the maceration method, these *Bacillus* species were found to be the only producers of the algal cell wall polysaccharidases tested for in this study. 

#### 2.7.3. Decaying *Ascophyllum nodosum* Isolated Using the ichip Method

Approximately 5% of the ichip-derived microbial communities screened were identified as being positive for one or more of HE-cellulose, lichenin, and pectin degrading activities. None of the bacterial isolates from 30 °C displayed MAPD activity under the conditions tested in this study while less than 3% of the 25 °C derived population tested positive and 15% from the 18 °C bacterial population were enzyme active. All the enzyme active bacterial isolates cultured from 18 °C were identified as belonging to the *Vibrio* genus. These isolates were found to produce pectin degrading enzymes (Table S1; Supplementary information).

### 2.8. Enzyme-Assisted Extraction (EAE) of Total Phenolics from *F. vesiculosus*

**Figure 3:**
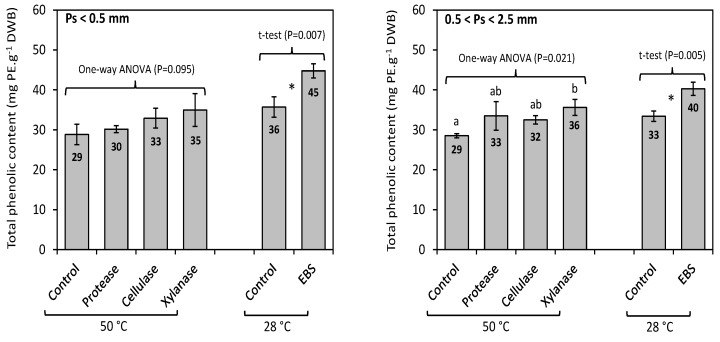
Enzymatic-assisted extraction of total phenolics from *Fucus vesiculosus* with commercial enzymes conducted at 50 °C, and with enzymatic bacterial supernatants (EBS) conducted at 28 °C. Control experiments without the addition of either commercial enzymes or EBS were conducted at 50 °C and 28 °C, respectively, under the same conditions. This experiment was undertaken using two different particle sizes (Ps) of ground biomass i.e., Ps < 0.5 mm, and 0.5 < Ps < 2.5 mm. Total phenolic content (TPC) is expressed as milligram of phloroglucinol equivalents (PE) per gram of dry weight biomass (mg PE.g^−1^ DWB). A one-way ANOVA was performed to assess significant differences (*p* < 0.05) between commercial enzymes and control (50 °C), results are arranged in increasing order: a < b; while a t-test was performed to determine significance differences (*p* < 0.05) between EBS and control (28 °C), asterisk (*) indicates a difference between both treatments.

We then compared the ability of an enzymatic bacterial supernatant (EBS) generated from the three isolates IC18_D7 (DSM 107285), IC18_D5 and ANT_0__A6 (DSM 107318) with ≥98% 16S rRNA gene sequence similarity to *Vibrio anguillarum* X0906, *Vibrio oceanisediminis* S37, and *Winogradskyella sp.* MGE_SAT_697, respectively, which we had selected as the best enzyme producers from our group of enzyme-active strains to perform enzyme-assisted extraction of phenolics from *Fucus vesiculosus*, and to compare their performance to commercially available enzyme preparations. Bacterial isolates IC18_D7 and IC18_D5 were shown to produce pectin degrading enzymes. ANT_0__A6 had previously been shown to produce good levels of amylase activity (data not shown). Results obtained from the EAE of the total phenolic compounds from *F. vesiculosus*, performed with or without commercial enzymes conducted at 50 °C, and with or without the enzymatic bacterial supernatants (EBS) conducted at 28 °C, are shown in Figure 3. The total phenolic content (TPC) of *F. vesiculosus* obtained by exhaustive solid-liquid extraction had previously been reported as 68.6 ± 8.3 mg PE.g^−1^ DWB [36]. This content was thus considered as a reference value for TPC, corresponding to a yield of extraction of 100%. Although the highest TPC values were obtained using commercial enzymes, compared to the control (50 °C), the increase was only significant when xylanase was used on the larger biomass particles i.e., 0.5 < Ps < 2.5 mm (*p* = 0.021). This TPC value of 35.6 ± 2.0 mg PE.g^−1^ DWB, obtained using xylanase, was equivalent to an extraction yield of 52%. Using the enzymatic bacterial supernatants (EBS), the TPC values increased significantly for both particle sizes (*p* < 0.01), compared to the control (28 °C), reaching up to 44.8 ± 1.8 mg PE.g^−1^ DWB (Ps < 0.5 mm) and 40.3 ± 1.7 mg PE.g^−1^ DWB (0.5 < Ps < 2.5 mm), respectively. These TPC values correspond to extraction yields of 65% and 59%, respectively. The extraction yields were therefore increased by 10% using xylanase, while they increased by 11–13% using EBS, compared to their respective controls. Moreover, an increase in extraction temperature (control 28 °C vs control 50 °C) appeared to have an overall negative effect on the extraction yield for phenolics. These results indicate that cell-wall degrading enzyme preparations produced by the three bacterial isolates from *A. nodosum*, applied at 28 °C were more efficient than the commercial protease, cellulase and xylanase preparations in the extraction of total phenolics from *F. vesiculosus*. 

## 3. Discussion

Macroalgal bioactive compounds are used in products to stimulate animal health or as functional food ingredients [28,37,38]. Phlorotannins exclusive to brown algae in high amounts (15% DW) have been shown to possess antidiabetic [39], antioxidant [40], and antiproliferative [41] effects. In addition, seaweed extracts are commonly used as biostimulants in agriculture [42] and have been proposed as a viable alternative protein crop for use in diets for monogastric livestock [43]. 

Seaweeds are well known to be associated with a diverse range of bacteria which colonize their nutrient-rich surfaces [13,21,22]. These bacteria are known to be a very good source of specific polysaccharidases, including pectinases, alginate lyases, carrageenanases, fucoidanases, and laminarinases [44] with several biotechnological applications. Some of these algal cell-wall degrading enzymes are produced to help mobilize polymers for nutritional purposes, for example, when growing in a nutrient limited state such as algal decay, and contribute to algal biomass recycling [20,21]. Thus, we reasoned that if *A. nodosum* was allowed to decay under controlled conditions at different temperatures, it should result not only in changes in the overall composition and dynamics of the bacterial communities present, but also in the isolation of bacteria that produce algal cell wall polysaccharidases, given the nutrient limited state to which they had been exposed, that might have potential application in EAE strategies.

The structure of the surface-attached bacterial population associated with intact and decaying *A. nodosum* incubated at 18 °C, 25 °C, and 30 °C was investigated in this study using both culture independent and culture dependent (traditional maceration and the in-situ cultivation based ichip device) approaches. The use of a next-generation sequencing approach (Illumina MiSeq) supplemented the 16rRNA gene-based approach employed on the cultured bacterial isolates. Given that the NGS approach circumvents the difficulties associated with the cultivation of bacteria from environmental samples and allows the identification of both cultivable and non-cultivable bacterial populations, it is not surprising that some phyla observed in the metagenomic communities of the macroalga were not identified in the total cultivable bacterial population. In particular, considering the isolation agar (SYP-SW) and the culture condition (72 h at 28 °C) employed in this study, it is highly unlikely that most phyla, including Planctomycetes, Spirochaetae, and Verrucomicrobia, which were found in the NGS dataset, would be recovered. These bacterial phyla would require a more targeted isolation strategy to be identified using various plate-based cultivation methods [45,46,47,48].

The ichip device, which has previously been reported to increase the microbial diversity of cultured bacterial isolates [49,50,51], was applied to potentially expand the range of bacterial isolates identified to include previously uncultured species. While the composition of the microbial communities derived from the ichip device did not differ greatly from the bacterial populations identified using the traditional approach (Figure 2), we recovered four potentially novel strains (IC25_B4, IC25_B12, IC25_C8, and IC25_G4) with 97% or less identity to their closest BLAST relative using the device. These bacterial isolates are currently being further characterized. The ichip device also resulted in the isolation of two strains (IC18_D5 and IC18_D7) identified as belonging to the *Vibrio* genus, extracts from which were subsequently utilized in the EAE of phenolics from *Fucus vesiculosus* and were found to be more efficient in the extraction process than current commercially available enzymes (Figure 3). This further demonstrates the utility of the ichip device as an important method to not only capture previously uncultivable bacteria, but also to recover bacteria with potential biotechnological applications [49,50].

Phylum-level analysis revealed that the structure of both the cultivable and metagenomic microbial communities found on the intact seaweed differed from that of the decaying macroalga, suggesting that the decay process plays a role in altering the algal associated microbial populations. However, these results should be interpreted with caution as our experiments were not conducted in replicates. Similar differences in the microbial community profiles associated with healthy and weakened bleached macroalgae have also been previously reported [52]. Although a causal link between such differences and the host condition has not been clearly established, it is known that host stress, such as bleaching and decay-related disruptions to the composition and abundance of its associated microbial consortium, can have detrimental effects on the host, causing diseases; for example, due to interferences with the seaweed–bacteria interactions that support algal development and host defense [18,53]. Chun et al. [20] suggest that microcosms which emerge as a result of the algal decay process may explain the differences in the bacterial populations associated with healthy and decaying algae. Decaying *Cladophora* samples have, for example, been shown to produce low oxygen and pH environments with increased ammonium-nitrogen levels. Subsequently, structural shifts in the microbial community towards bacterial groups better suited to thrive under such conditions were observed [20]. While the succession of oxygen concentration, pH and nutrient levels during the decay period was not monitored in this study, the structural shifts observed in the microbial communities with decay may be attributed to changes in the composition of the closed microcosm within the shake flasks. 

Screening the cultivable surface microbiota of both the intact and decaying seaweed for the production of algal cell wall polysaccharide degrading enzymes revealed a number of MAPD bacteria. Bacteria belonging to the genus *Bacillus* which represented the major producers (>80%) of these hydrolytic enzymes were not identified in the bacterial population associated with *A. nodosum* in its healthy state but represented up to 10% of the surface microbiota communities isolated during the algal decay (Figure S1; Supplementary information). This marked difference in the composition and abundance of the microbial communities associated with the seaweed during its different physiological states (intact and decaying), mainly characterized by the emergence in the members of the enzymatically active *Bacillus* and *Vibrio* groups supports the hypothesis that nutrient limiting conditions such as algal decay is likely to promote the proliferation of MAPD producing bacteria [21]. However, while the number of MAPD isolates was not observed to steadily increase during decay as might be expected due to the weakened state of the seaweed, the few enzymatically active strains that we did identify during decay, such as the *Bacillus* and *Vibrio* species were efficient producers of the MAPD enzymes for which we tested (Table S1; Supplementary information). 

Microorganisms are well-known to exhibit mutualism such that one or more individuals within a microbial population can gain from the collective characteristics expressed by its neighbors without expressing the trait itself [14,54,55]. Such phenotypically deficient bacteria may however possess the metabolic capability necessary to utilize nutrients provided by other members of the community [55]. A lack in the increase in the expected numbers of MAPD bacteria that we observed during the decay experiments may thus be explained by the efficiency of the less abundant enzymatically active strains who may be compensating for the inactivity of the dominant species by creating a pool of available nutrients thereby supporting the overall bacterial consortia present within the microcosm in the growth flasks, to which no nutrients had been added.

Finally, we assessed the ability of enzymatic bacterial supernatant (EBS) from a selected group of enzyme-active strains; IC18_D7, IC18_D5 and ANT_0__A6 with similarity to *Vibrio anguillarum* X0906, *Vibrio oceanisediminis* S37, and *Winogradskyella sp.* MGE_SAT_697, respectively, in the enzyme-assisted extraction of phenolics from *Fucus vesiculosus*. These enzyme preparations were shown to increase total phenolic content (TPC) extraction yields from *Fucus vesiculosus* by 11–13%, to levels which were greater than the extraction yields obtained using a commercially available xylanase (10%) (Figure 3). To our knowledge, this is the first study to report the application of macroalgal-derived bacterial culture extracellular supernatants in the enzyme-assisted extraction of phenolics from *Fucus vesiculosus.* Thus, it is clear that bacterial populations associated with *A. nodosum* are a good source of algal cell wall polysaccharide degrading enzymes with potential utility in EAE strategies. The isolation of macroalgal associated bacteria is frequently reported in the literature [20,21], with isolates being developed for use in various biotechnological applications, such as novel carrageenanases from *Flavobacteria* and γ-Proteobacteria isolated from *Ascophyllum nodosum* [21] and from *Pseudoalteromonas porphyrae* isolated from decayed seaweed [56] for potential biomedical and food applications, together with alginate lyase from *Zobellia galactanivorans* for biomass degradation [57]. Our study further demonstrates the potential utility of algal derived bacteria and their potential contribution to EAE based strategies aimed at the production of seaweed extracts for similar types of biotechnological applications.

## 4. Materials and Methods

### 4.1. Sampling 

*Ascophyllum nodosum* samples were obtained in the intertidal zone at Rinville in Galway Bay, Ireland at 53°14′40′′ North, 8°58′2′′ West in late January, 2016. Approximately 2 kg of seaweed was sampled and packaged in sterile air-tight plastic bags and stored on dry ice at the sampling location. *Ascophyllum* samples were subsequently stored briefly at 4 °C in the laboratory before further analyses. 

### 4.2. Experimental Design

Three sets of approximately 450 g of the seaweed were suspended in separate 950 mL sterile artificial seawater (3.33% *w*/*v* synthetic seawater salts Instant Ocean, Aquarium Systems, in distilled water), with each 2 L flask incubated at a different temperature (18 °C, 25 °C, and 30 °C) on a shaking platform at 125 rpm for a six-week period. The incubation flasks were single replicates (*n* = 1) per temperature treatment. Three separate ichip devices were subsequently inoculated four weeks after the initial incubation into each of the flasks under sterile conditions in a laminar flow hood (BioAir Safeflow 1.2 – EuroClone, Pero, Italy), as previously described [32], and removed at the end of the decay period. To inoculate each ichip device, 1 mL suspension from the incubation flask containing the seaweed decaying at one of the three temperatures (18 °C, 25 °C, and 30 °C), with an estimated microbial density of 1.0 × 10^12^ cells mL^−1^ was diluted appropriately in sterile artificial seawater (3.33% *w*/*v* synthetic seawater salts Instant Ocean, Aquarium Systems, in distilled water) to attain an average of one cell per through-put hole in the iChip central plate (i.e., one cell per µL of inoculum) and suspended in molten 0.5% agar (Sigma Aldrich, Munich, Germany) solution. The cell-agar suspension was poured over the ichip central plate to allow cells that were immobilized within the suspension to be trapped in the small throughput holes on the device as the agar solidified. The device was then assembled and placed in the flask containing the decaying seaweed for another two weeks. Approximately 10 g of the fresh intact macroalga (T_0_) was collected before incubation into the artificial seawater and 10 g of the decaying seaweed was collected from each incubating flask at two-week intervals (weeks 2, 4, and 6) during the decay period. All *Ascophyllum* samples were stored at −20 °C for further analyses.

### 4.3. 16S rRNA Gene Amplicon Library Preparation and MiSeq Sequencing

Metagenomic DNA was extracted from approximately 0.5 g of the intact seaweed (T_0_) and 0.5 g each of decaying *Ascophyllum nodosum* samples collected at three phases of the decay period (week 2, week 4 and week 6) at 18 °C (2_18, 4_18, 6_18), 25 °C (2_25, 4_25, 6_25), and at 30 °C (2_30, 4_30, 6_30) as previously described [58]. PCR amplicon libraries were generated using forward (5′ *TCGTCGGCAGCGTCAGATGTGTATAAGAGACAG*CCTACGGGNGGCWGCAG-3′) and reverse (*GTCTCGTGGGCTCGGAGATGTGTATAAGAGACAG*GACTACHVGGGTATCTAATCC-3′) primers complementary to the V3-V4 16S rRNA gene region [59] with ligated Illumina adapter overhang sequences in italic text. This primer pair was identified as the most promising pair required for a good representation of bacterial diversity and has been successfully applied in a number of studies on a wide range of environments [59,60,61,62]. PCR amplification was performed under the following conditions: 98 °C for 30 s, followed by 30 cycles of denaturation (98 °C for 10 s), primer annealing (57 °C for 30 s), primer extension (72 °C for 30 s), and 72 °C for 5 min. PCR amplicons were purified using Agencourt AMPure XP beads (Beckman Coulter) according to the manufacturer’s instructions and a subsequent reduced-cycle (8 cycles) reaction was performed to further attach unique dual eight-base Nextera XT multiplexing indices and sequencing adapters under similar cycling conditions. Index PCR products were purified using Agencourt AMPure XP beads (Beckman Coulter; Fisher Scientific, Dublin, Ireland) according to the manufacturer’s instructions. All PCR reactions from each sample were performed in triplicates to minimize bias, replicate amplicons were pooled together and sequenced using the Illumina MiSeq platform by Macrogen (Seoul, Korea).

Scythe (v0.994 BETA) [63] and Sickle [64] programs were used to quality trim raw reads and remove adapter sequences. This service was provided by Macrogen Inc (Seoul, Korea) as part of a next generation sequencing package. Trimmed paired end reads were merged (using the join_paired_reads.py script with the fastq-join method [65] ) in QIIME version 1.9.1 (QIIME.org) [34] and processed using standard QIIME version 1.9.1 protocols (http://qiime.org/tutorials/illumina_overview_tutorial.html). Briefly, a further quality step was applied by excluding reads with a Phred score less than 20 using the split_libraries_fastq.py QIIME script. The USEARCH algorithm [66] was used to remove chimeras and assign sequences to OTUs based on the SILVA database (version 123) (Max Plank Institute, Bremen, Germany) [35] at a threshold of 97% identity. Singletons were identified and filtered from the OTU table and the OTU table was CSS (cumulative sum scaling) normalized [67]. The taxonomy identified from the dataset was then represented through bar plots. Species diversity and richness within samples were calculated using alpha and beta diversity analyses (Chao1, Good’s coverage, Shannon indices and principle coordinates analysis) using QIIME (version 1.9.1) (http://qiime.org/tutorials/illumina_overview_tutorial.html) scripts (alpha_diversity.py and beta_diversity_through_plots.py) [34].

### 4.4. Bacterial Isolation from Intact and Decaying A. nodosum Using Maceration Method

Surface-attached bacteria were isolated from the intact (T_0_) and decaying seaweed samples collected at weeks 2, 4 and 6 of the decay period, each at three different temperatures (18 °C, 25 °C, and 30 °C) using the maceration method adapted from [68]. Briefly, approximately 0.5 g of the algal sample was cut into small pieces of about 1 cm^2^ and suspended in 1 mL of sterile artificial seawater (3.33% *w*/*v* synthetic seawater salts Instant Ocean, Aquarium Systems, in distilled water) [69]. Serial dilutions of the suspension were plated on SYP-SW agar plates which consisted of soluble starch (Sigma Aldrich, Munich, Germany) 10 g L^−1^; yeast extract (Sigma Aldrich, Germany) 4 g L^−1^; peptone (Merck, Germany) 2 g L^−1^; Instant Ocean (Aquarium Systems) 33.3 g L^−1^; agar (Sigma Aldrich, Germany) 15 g L^−1^ and incubated at 28 °C for 72 h. The culture isolation procedure was conducted aseptically in a laminar flow hood (BioAir Safeflow 1.2—EuroClone, Pero, Italy). Individual colonies were selected and further streaked to isolate pure cultures which were grown at 28 °C overnight in SYP-SW medium and maintained in glycerol (20% *w*/*v*) stocks at −80 °C.

### 4.5. Bacterial Isolation from Decaying A. nodosum Using ichip Device

Three separate ichip devices were inoculated into each of the incubating flasks (18 °C, 25 °C, and 30 °C) at week 4 of the decay period and were removed at the end of the decay period (week 6). Macroalgal-associated bacteria from decaying *A. nodosum* were recovered from small throughput holes on the central plate of each ichip device and plated directly onto 96-well plates containing SYP-SW agar and incubated at 28 °C for 72 h. Individual colonies were selected and further streaked to isolate pure cultures which were grown at 28 °C overnight in SYP-SW medium and maintained in glycerol (20% *w*/*v*) stocks at −80 °C. 

### 4.6. Taxonomic Identification of A. nodosum Cultivable Surface Microbiota Populations 

Bacterial isolates recovered from both intact and decaying *A. nodosum* samples using both the traditional maceration method and the ichip method were taxonomically identified using 16S rRNA gene sequencing. Genomic DNA was extracted from the bacterial isolates grown overnight at 28 °C in SYP-SW medium using a modified Tris-EDTA boiling DNA extraction method [70]. Bacterial 16S rRNA PCR amplification was performed with the universal forward (8F; 5′-AGAGTTTGATCCTGGCTCAG-3′ or 27F; 5′-AGAGTTTGATCMTGGCTCAG-3′) and reverse (1492R; 5’-GGTTACCTTGTTACGACTT-3′) primers [71,72] under the following conditions: initial denaturation (95 °C for 30 s), followed by 35 cycles of denaturation (95 °C for 1 min), primer annealing (55 °C for 1 min), primer extension (72 °C for 1 min) and a final primer extension step (72 °C for 5 min). PCR products were analyzed by gel electrophoresis on a 1% agarose gel and purified using a QIAquick PCR Purification Kit (Qiagen, Hilden, Germany) according to the manufacturer’s instructions.

Sanger sequencing was performed on the amplified PCR products by GATC Biotech, (Konstanz, Germany) and Macrogen (Amsterdam, The Netherlands). Low quality 5’ and 3’ sequence ends were trimmed using FinchTV (http://www.geospiza.com/finchtv) depending on the data set. The BLAST program (NCBI) (https://blast.ncbi.nlm.nih.gov/Blast.cgi) was used to compare trimmed sequences against the GenBank database and closest relatives to the bacterial isolates were identified. 16S rRNA gene sequences were checked for chimeras using USEARCH algorithm [66] and the data sets were de-replicated using the Fastgroup database [73] and Avalanche NextGen Workbench version 2.30 (http://www.visualbioinformatics.com/html/)(bioinformatics.org) with a 99% cut-off value. Sequence alignment and phylogenetic tree construction were performed with MEGA (version 7) (Penn State University, PA, USA) [74]. The evolutionary history was inferred using the Neighbor-joining method [75]. 

### 4.7. Enzyme Screens

Bacterial isolates obtained from both intact *A. nodosum* and the decaying seaweed, using both the maceration method and the ichip device, were screened for the production of macroalgal cell wall degrading enzymes including pectinase, hydroxyethyl cellulase, and enzymes involved in lichenin degradation. Bacterial isolates were grown at 28 °C for 72 h on LB gellan gum (Sigma Aldrich, Munich, Germany) plates supplemented with the appropriate substrate, at a concentration of 0.2% (*w*/*v*) for pectin (Sigma Aldrich, Germany), 0.5% (*w*/*v*) for hydroxyethyl cellulose (Sigma Aldrich, Germany) and 0.05% (*w*/*v*) for lichenin (Megazyme). Enzymatic activities on lichenin and HE-cellulose were indicated by a surrounding zone of clearance upon flooding with Congo red solution (0.1% *w*/*v* Congo red in 20% *v*/*v* ethanol) for 30 min and wash with 1M NaCl for 5 min [76,77] while pectin supplemented plates were flooded with Lugol’s iodine solution [78]. 

### 4.8. Enzyme-Assisted Extraction

Entire specimens of *Fucus vesiculosus* (2–3 kg fresh weight) were collected at low tide on 6 July 2016 at Finavarra, Co. Clare, Ireland (53°08′59″ North–9°08′09″ West). In the laboratory, on the day of collection, algal biomass was cleaned of epiphytes and rinsed in distilled water to remove excess salt. Samples were patted dry with tissue paper and stored at −20 °C. Then, the biomass was freeze-dried in a Labconco Freezone^®^ freeze-dryer system (Labconco Corporation, Kansas City, MO 64132-2696, USA). Dried biomass was ground using a coffee-grinder and sieved to produce two types of powder, Ps < 0.5 mm and 0.5 < Ps < 2.5 mm), prior to subsequent enzymatic-assisted extraction (EAE) of total phenolic compounds. Three commercially available enzymes; cellulase (from *Aspergillus* sp., Sigma Aldrich, ≥1000 U/g), xylanase (from *Trichoderma* sp., Megazyme, 2.86 U/mL) and protease (from *Bacillus licheniniformis*, Sigma Aldrich, ≥2.4 U/g) were used for the hydrolysis of the seaweed. The potential of three bacterial strains (IC18_D7, IC18_D5 and ANT_0__A6 with ≥ 98% sequence similarity to *Vibrio anguillarum* X0906, *Vibrio oceanisediminis* S37 and *Winogradskyella* sp. MGE_SAT_697, respectively) isolated from *A. nodosum* and shown to be producers of algal cell wall degrading enzymes in this study was also tested. Bacterial isolates were grown overnight at 28 °C in SYP-SW medium and equal volumes of supernatants from the overnight cultures obtained by centrifugation at 4300× *g* for 10 min were used. Three sets of approximately 4 g dry weight of the crushed algae was incubated at 50 °C for 24 h on a shaking platform (185 rpm) with sodium acetate buffer (100 mL; 0.1 M; pH 5.2), each with 100 µL of one of the three commercial enzymes. Another set of the algal biomass was also incubated at 28 °C with a mixture of culture supernatants obtained from the bacterial isolates (enzymatic bacterial supernatants, EBS) to a final volume of 100 µL. All experiments were performed in triplicate and control experiments without the addition of either commercial enzymes or culture supernatants (EBS) were also conducted under the same conditions. The hydrolysate mixture from each experimental set was centrifuged at 4300× *g* for 10 min at 4 °C to eliminate the algal debris from the extract. The different extracts produced were freeze dried, weighed, and stored at −80 °C until further analysis for total phenolics.

### 4.9. Determination of Total Phenolic Content (TPC)

The total phenolic content (TPC) of the *F. vesiculosus* crude extracts was determined using a slightly modified version of the Folin–Ciocalteu assay [79] as described by [80]. A known amount of crude extract was re-suspended in methanol to a concentration of 1 mg.mL^−1^. 100 μL of each crude extract was placed in a 1.5 mL Eppendorf tube along with 100 μL of methanol, 100 μL of Folin–Ciocalteu reagent (2N) and 700 μL of 20% sodium carbonate, to a final volume of 1 mL. Samples were vortexed and immediately afterwards placed in darkness to incubate for 20 min at room temperature. Samples were then centrifuged at 4300× *g* for 3 min before measuring the absorbance of the supernatant at 735 nm using a Cary UV50 Spectrophotometer and CaryWIN software (Varian Inc., Palo Alto, CA 94304, USA). A sample treated according to the same protocol, but where 100 µL methanol instead of 100 µL of crude extract (1 mg·mL^−1^) was added, was used as a blank. Phloroglucinol was used as the external standard and a calibration curve was performed by serial dilution of a 2 mg mL^−1^ stock solution (10, 20, 50, 80, 120, 160 μg mL^−1^). Total phenolic content (TPC) was expressed as milligram of phloroglucinol equivalents (PE) per gram of dry weight extract (mg PE g^−1^ DWE) or per gram of dry weight biomass (mg PE g^−1^ DWB) [36]. TPC quantification was performed in triplicate for each crude extract. The yield of extraction was calculated after exhaustive solid-liquid extraction (i.e., three successive extraction) of the total phenolics of 50 mg of *F. vesiculosus* freeze-dried ground biomass (Ps < 0.5 mm) using 80% methanol.

### 4.10. Accession Numbers

The metagenomic sequencing data (raw reads) was deposited in the European Nucleotide Archive (ENA) under the accession numbers ERR2608102 -ERR2608111. The 16S rRNA gene sequences for the bacterial isolates were deposited in GenBank under the accession numbers KY224981–KY225289, KY327837, KY327838, MG693225–MG693716, MG760723–MG760725, and MK480287–MK480325. Bacterial isolates IC18_D7 and ANT_0__A6 with enzymatic activities and applicability in EAE approach were deposited in DSMZ culture collection bank under the accession numbers DSM 107285 and DSM 107318, respectively.

### 4.11. Statistical Analysis

Prior to performing statistical analyses on data obtained by enzyme-assisted extraction (EAE) of total phenolics from *F. vesiculosus*, tests of normality were carried out with the Kolmogorov–Smirnov test for normal distributions and Levene’s test for homogeneity of variance. A one-way ANOVA and post hoc Tukey’s pairwise test was performed to assess significant differences (*p* < 0.05) between commercial enzymes and control (50 °C); and a *t*-test was applied to assess significance differences (*p* < 0.05) between EBS and control (28 °C). All data treatments and statistical analyses were performed using IBM SPSS Statistics V22.0 (IBM Corporation, Armonk, NY, USA).

## 5. Conclusions

In conclusion, we have demonstrated, using both metagenomic and culture based approaches, that changes occur in the composition and abundance of *A. nodosum*-associated epibiotic communities which are both time and temperature dependent and that the microflora of *A. nodosum* is composed of diverse and complex bacterial communities which produce a wide range of hydrolytic enzymes, some of which may be useful in future EAE based strategies in the agricultural, food, cosmeceutical, and pharmaceutical sectors. 

## Figures and Tables

**Table 1:** Observed OTUs and species richness and diversity estimates of *Ascophyllum*-associated metagenomic communities obtained using MiSeq sequencing of the 16S rRNA gene from the intact seaweed (T_0_) and each of decaying *Ascophyllum nodosum* samples collected at three phases of the decay period (week 2, week 4 and week 6) at 18 °C (2_18, 4_18, 6_18), 25 °C (2_25, 4_25, 6_25), and at 30 °C (2_30, 4_30, 6_30).

Decay Period	Sample	No. of Reads after Quality Filtering	No. of OTUs (at 97% Sequence Identity)	Chao1 Richness	Shannon Index
**Week 0** **intact seaweed**	T_0_	178,699	1467	2293.5	10.1
**Week 2** **early decay phase**	2_18	123,551	854	2072.1	9.5
	2_25	350,135	1476	3327.1	10.2
	2_30	138,445	724	1822.7	9.2
**Week 4** **mid decay phase**	4_18	148,904	749	1901.7	9.3
	4_25	151,285	1130	2061.6	9.8
	4_30	139,737	1633	3490.4	10.4
**Week 6** **late decay phase**	6_18	120,679	443	1148	8.6
	6_25	138,659	1202	2884.5	10.0
	6_30	165,816	1250	3062.8	10.0

**Table 2:** Number of bacterial isolates cultured from intact (week 0) and decaying *Ascopyllum nodosum* samples (week 2, week 4, and week 6), incubated at different temperatures, using the maceration isolation method and the ichip device.

Week 0	Week 2	Week 4	Week 6	ichip Isolation	Incubating Temperature
**90**	76	63	52	59	18 °C
	35	47	70	76	25 °C
	63	67	53	89	30 °C

## Supplementary Materials

The following are available online at https://www.mdpi.com/1660-3397/17/4/200/s1; Table S1: Table showing enzymatic activities, Figure S1: *Ascophyllum* cultivable surface microbiota at genus classification level, Figures S2–S6; Phylogenetic trees of intact and decaying *A. nodosum* associated microbial communities.
