# Supplementary material for: Microbial Population Changes in Decaying Ascophyllum nodosum Result in Macroalgal-Polysaccharide-Degrading Bacteria with Potential Applicability in Enzyme-Assisted Extraction Technologies
Source: Mar Drugs. 2019 Mar 29;17(4):200. doi: 10.3390/md17040200 (PMC6520818; doi:10.3390/md17040200)
Supplement: Supplementary file 1 [file marinedrugs-17-00200-s001.pdf]

marine drugs

Article

Microbial population changes in decaying *Ascophyllum nodosum* result in macroalgal-polysaccharide-degrading bacteria with potential applicability in enzyme-assisted extraction technologies.

Maureen W. Ihua[1], Freddy Guihéneuf[2], Halimah Mohammed[1], Lekha M. Margassery[1], Stephen A. Jackson[1], Dagmar B. Stengel[3], David J. Clarke[1,4], Alan D.W. Dobson[1,5 *]

[1]School of Microbiology, University College Cork, Cork, Ireland; w.ihua@umail.ucc.ie (M.I.); halimahmoh8@gmail.com (H.M.); lekha513@gmail.com (L.M.); sjackson@ucc.ie (S.J.); a.dobson@ucc.ie (A.D.)

[2]Laboratoire d'Océanographie de Villefranche-sur-Mer (LOV) France; freddy.guiheneuf@obs-vlfr.fr (F.G.)

[3]Botany and Plant Science, School of Natural Sciences, Ryan Institute for Environmental, Marine and Energy Research, National University of Ireland Galway, Galway, Ireland; dagmar.stengel@nuigalway.ie (D.S.)

[4]APC Microbiome Institute, University College Cork, Cork, Ireland; david.clarke@ucc.ie (D.C.)

[5]Environmental Research Institute, University College Cork, Cork, Ireland; a.dobson@ucc.ie (A.D.)

[*]Correspondence: a.dobson@ucc.ie (A.D.)

Keywords: *Ascophyllum nodosum*, algal cell wall degrading enzymes, enzyme-assisted extraction, iChip device

marine drugs

MDPI

Legends to Supplementary figures

Table S1

Ascophyllum nodosum associated bacterial isolates, their closest BLAST relative and observed enzymatic activities. Bacterial strains were examined for their hydroxyethyl cellulose (HE-cellulase), lichenase and pectinase activities. Enzymatic activity is indicated by a (+) sign while a (-) sign indicates that no enzymatic activity was observed under the conditions tested

Figure S1

Relative abundances at genus level of bacteria associated with the cultivable surface microbiota of (a) intact *Ascophyllum nodosum* and decaying *Ascophyllum nodosum* at 2, 4 and 6 weeks of decay at (a) 18 °C; 2_18, 4_18, 6_18 (b) 25 °C; 2_25, 4_25, 6_25 (c) 30 °C; 2_30, 4_30, 6_30 which were obtained by maceration culture isolation method and (e) obtained by ichip culture isolation method. 16S rRNA gene sequences were obtained from the bacterial isolates and taxonomic analyses were performed. The relative distribution of phyla in each group is represented as a percentage

Figure S2

Neighbor-joining phylogenetic tree representing bacterial phyla cultured from *Ascophyllum nodosum* sample before induced decay (T_0). The evolutionary relationships of each phylum identified are shown with reference sequences from NCBI included. This phylogenetic analysis was performed using single representative 16S rDNA sequences from each group

marine drugs MDPI

identified by Fastgroup program. The number of similar sequences represented by each sequence is shown in brackets. This tree was drawn using MEGA program (version 7) and bootstrapping percentages (1000 replicates) above 50% are shown

Figure S3

Neighbor-joining phylogenetic tree representing bacterial phyla cultured from *Ascophyllum nodosum* sample at week 2 of induced decay from 18 ºC, 25 ºC and 30 ºC. The evolutionary relationships of each phylum identified are shown with reference sequences from NCBI included. This phylogenetic analysis was made using single representative 16S rDNA sequences from each group identified by Avalanche NextGen Workbench version 2.30. The number of similar sequences represented by each sequence is shown in brackets. This tree was drawn using MEGA program (version 7) and bootstrapping percentages (1000 replicates) above 50% are shown

Figure S4

Neighbor-joining phylogenetic tree representing bacterial phyla cultured from *Ascophyllum nodosum* sample at at week 4 of induced decay from 18 ºC, 25 ºC and 30 ºC. The evolutionary relationships of each phylum identified are shown with reference sequences from NCBI included. This phylogenetic analysis was made using single representative 16S rDNA sequences from each group identified by Avalanche NextGen Workbench version 2.30. The number of similar sequences represented by each sequence is shown in brackets. This tree was drawn using MEGA program (version 7) and bootstrapping percentages (1000 replicates) above 50% are shown.

marine drugs

MDPI

Figure S5

Neighbor-joining phylogenetic tree representing bacterial phyla cultured from *Ascophyllum nodosum* sample at the end of the decay period (week 6) from ● 18 °C, ■ 25 °C and 30 °C ▲ .The evolutionary relationships of each phyla identified are shown with reference sequences from NCBI included. This phylogenetic analysis was made using single representative 16S rDNA sequences from each group identified by Avalanche NextGen Workbench version 2.30. The number of similar sequences represented by each sequence is shown in brackets. This neighbor joining tree was drawn using MEGA program (version 7) and bootstrapping percentages (1000 replicates) above 50% are shown.

Figure S6

Neighbour-joining phylogenetic tree representing bacterial phyla cultured from ● 18 °C, ■ 25 °C and ▲ 30 °C using the iChip device. The evolutionary relationships of each phylum identified are shown with reference sequences from NCBI included. This phylogenetic analysis was performed using single representative 16S rDNA sequences from each group identified by Fastgroup. The number of similar sequences represented by each sequence is shown in brackets. This neighbor joining tree was drawn using MEGA program (version 7) and bootstrapping percentages (1000 replicates) above 50% are shown

marine drugs

MDPI

Table S1

SAMPLE ID	TOP BLAST HIT	IDENTITY (%)	ALGAL CELL WALL POLYSACCHARIDE DEGRADING ACTIVITIES		
			HE-cellulase	Lichenase	pectinase
**AN218_A2**	*Bacillus safensis* strain Rb1S1	100	-	+	-
**AN218_H5**	*Bacillus* sp. M101(2010) strain M101	100	+	+	-
**AN225_A5**	*Bacillus altitudinis* strain CT10	99	-	+	-
**AN225_A11**	*Bacillus licheniformis* strain HQB814	99	+	-	+
**AN225_B8**	*Bacillus licheniformis* strain AG-06	100	-	-	+
**AN225_B9**	*Bacillus licheniformis* strain ST7	99	-	-	+
**AN225_C1**	*Bacillus pumilus* strain ASpB9	99	-	+	-
**AN225_C7**	*Bacillus aerius strain* APBSMLB109	99	-	+	-
**AN225_C11**	*Bacillus* sp. 11RB3	99	-	+	+
**AN225_D1**	*Bacillus licheniformis* strain APBSWPTB167	100	+	-	+
**AN225_D4**	*Bacillus subtilis* strain HDXJ04	99	+	+	+
**AN225_D6**	*Bacillus pumilus* strain ASpB9	100	-	+	-
**AN225_E1**	*Bacillus pumilus* strain ASpB9	100	-	+	-
**AN225_E6**	*Bacillus licheniformis* strain JMB003	99	-	-	+
**AN225_E7**	*Bacillus licheniformis* strain V24	100	-	-	+
**AN225_E8**	*Bacillus licheniformis* strain V24	100	-	-	+
**AN225_E9**	*Bacillus* sp. strain SKS7	99	-	-	+
**AN225_E10**	*Bacillus licheniformis* strain KB102	99	-	-	+
**AN225_E11**	*Bacillus pumilus* strain ASpB9	100	-	+	-
**AN225_F6**	*Bacillus* sp. strain 703	100	-	-	+
**AN225_F9**	*Bacillus* sp. strain C60	99	-	+	
**AN225_F12**	*Bacillus licheniformis* strain V24	100	+	-	+

**AN225_G2**	*Bacillus* sp. strain SKS7	100	+	-	+
**AN225_G3**	*Bacillus* sp. (in: Bacteria) strain VI/7	100	-	-	+
**AN225_G6**	*Bacillus subtilis* strain AKKVG-2-18	100	-	+	+
**AN225_G8**	*Bacillus* sp. (in: Bacteria) strain VI/7	100	-	-	+
**AN230_A10**	*Bacillus pumilus* strain ASpB9	100	-	+	-
**AN230_B4**	*Bacillus* sp. strain SKS7	100	+	-	+
**AN230_B11**	*Bacillus mycoides* strain LBUM203	99	+	-	+
**AN230_D9**	*Bacillus licheniformis* strain V24	100	+	-	+
**AN230_D11**	*Bacillus licheniformis* strain V24	100	+	-	+
**AN230_E3**	*Bacillus* sp. (in: Bacteria) strain V52	100	-	-	+
**AN230_E4**	*Bacillus* sp. Ph_25A	100	-	+	-
**AN425_D9**	*Bacillus* sp. strain CZL003	100	+	+	+
**AN425_D11**	*Bacillus licheniformis* strain 8B-B92	99	+	-	+
**AN425_D12**	*Bacillus* sp. strain SKS7	100	+	-	+
**AN425_E4**	*Bacillus* sp. strain BS155	100	+	+	+
**AN425_G7**	*Bacillus* sp. strain BS155	100	+	-	+
**AN618_A1**	*Bacillus pumilus* strain ASpB9	100	-	+	-
**AN618_A2**	*Bacillus pumilus* strain ASpB9	100	-	+	-
**AN618_B10**	*Bacillus pumilus* strain ASpB9	100	-	+	-
**AN618_D11**	*Bacillus pumilus* strain ASpB9	100	+	+	-
**AN618_H4**	*Bacillus pumilus* strain ASpB9	100	-	+	-
**AN625_A10**	*Bacillus pumilus* strain ASpB9	100	-	+	-
**AN625_D7**	*Bacillus pumilus* isolate TD22	100	-	+	-
**AN625_G10**	*Bacillus pumilus* strain ASpB9	100	-	+	-
**AN630_A12**	*Bacillus hwajinpoensis* strain 16E11	99	-	+	-
**AN630_D1**	*Bacillus pumilus* strain ASpB9	100	-	+	-
**AN630_D2**	*Bacillus pumilus* strain ASpB9	100	-	+	-
**AN630_G12**	*Bacillus pumilus* strain ASpB9	100	-	+	-
**AN630_H8**	*Bacillus safensis* strain Rb1S1	100	-	+	-
**IC18_D5**	*Vibrio oceanisediminis* strain S37	98	-	-	+
**IC18_D6**	*Vibrio anguillarum* strain INTA11	100	-	-	+
**IC18_D7**	*Vibrio anguillarum* strain X0906	99	-	-	+

**IC18_D8**	*Vibrio oceanisediminis* strain S37	99	-	-	+
**IC18_D9**	*Vibrio anguillarum* strain X0906	99	-	-	+
**IC18_E2**	*Vibrio oceanisediminis* strain S37	98	-	-	+
**IC18_E6**	*Vibrio anguillarum* strain KAP1	100	-	-	+
**IC18_E7**	*Vibrio oceanisediminis* strain S37	99	-	-	+
**IC18_E8**	*Vibrio anguillarum* strain INTA11	100	-	-	+
**IC25_C11**	*Micrococcus yunnanensis*	100	-	-	+
**IC25_F10**	*Micrococcus yunnanensis*	100	-	-	+

Figure S1

marine drugs

MDPI

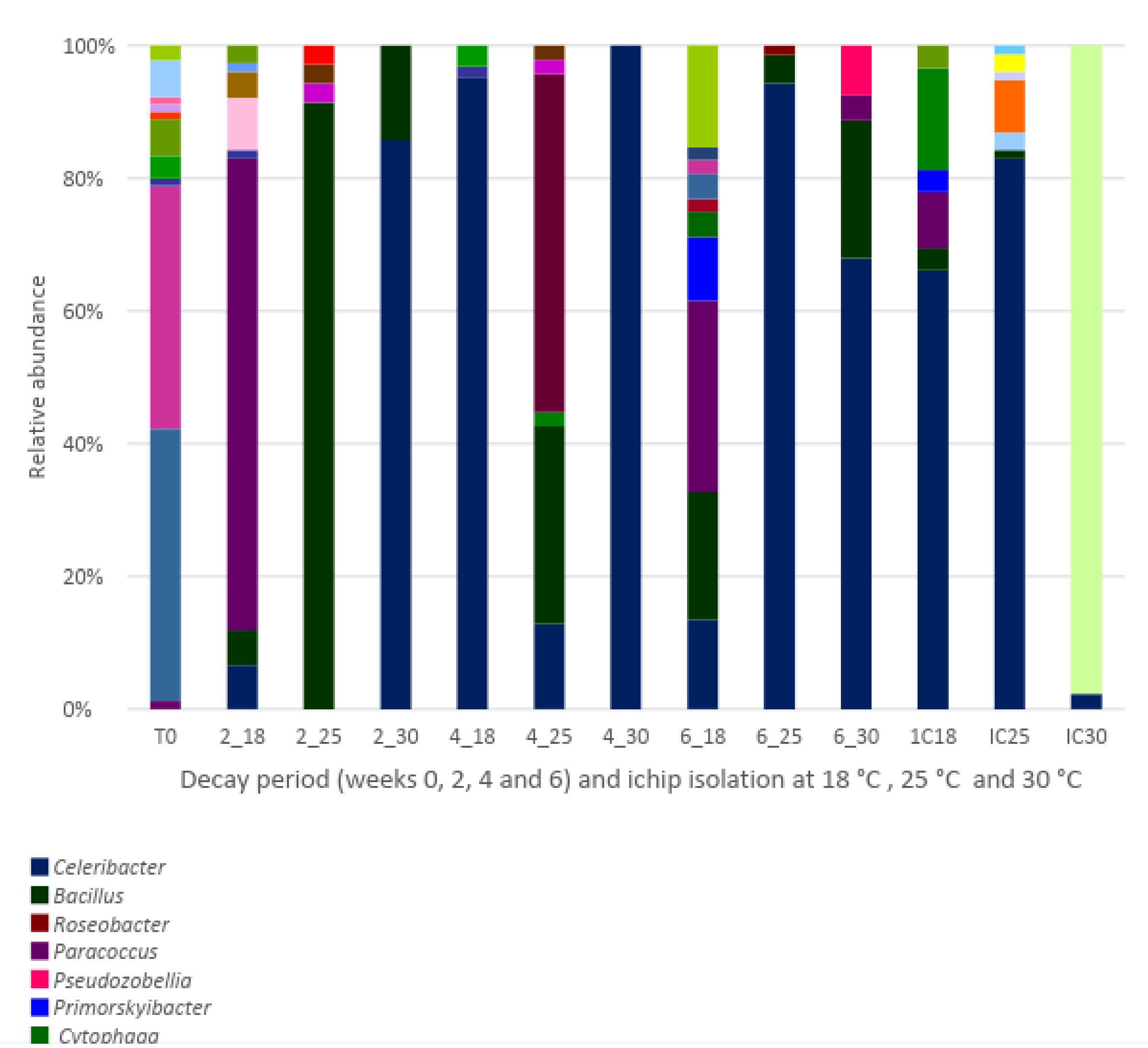

marine drugs

MDPI

Figure S2

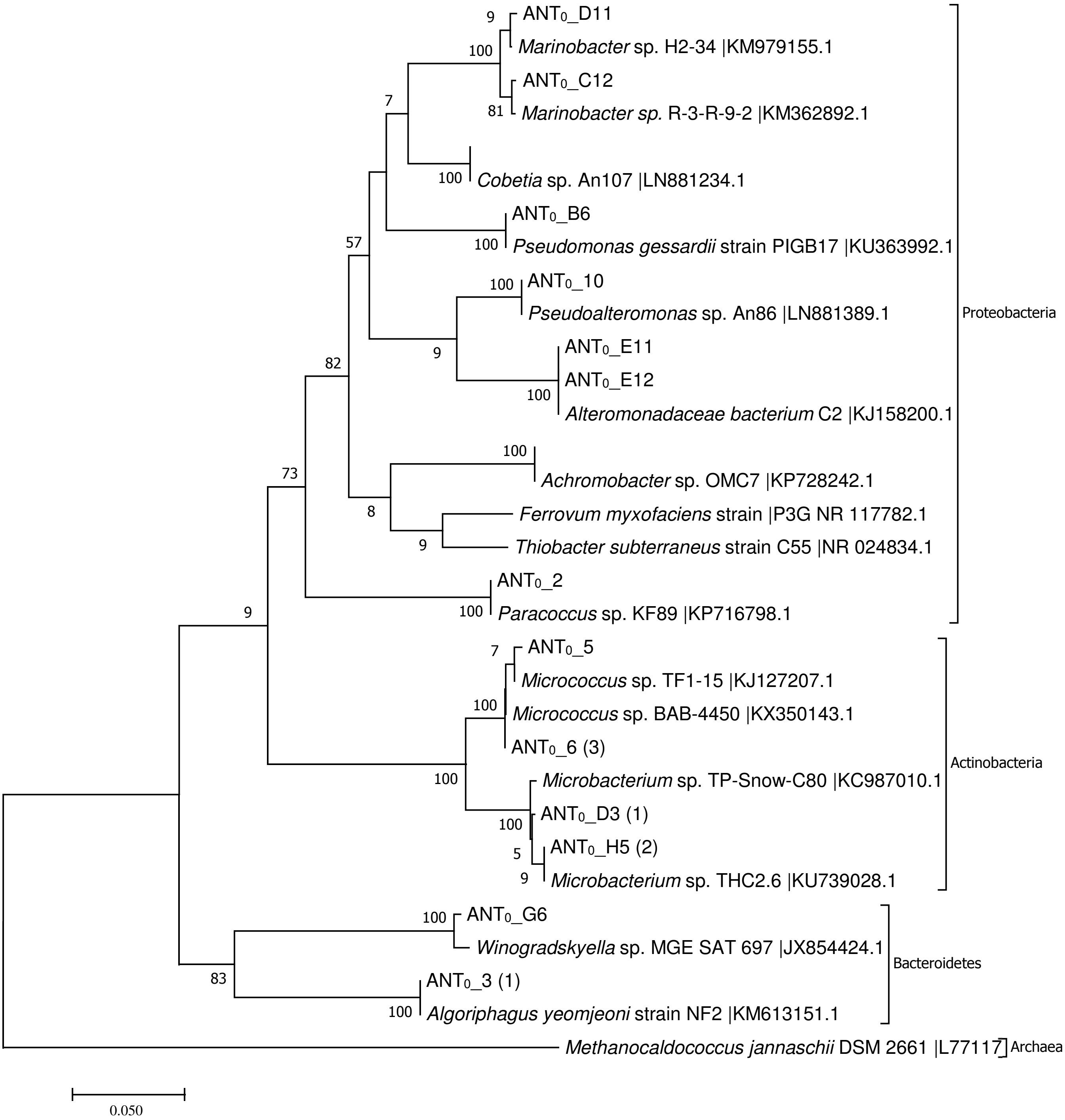

Figure S3

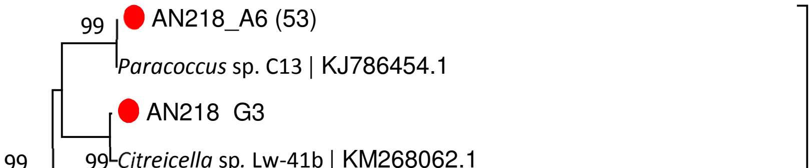

marine drugs

MDPI

Figure S4

marine drugs

MDPI

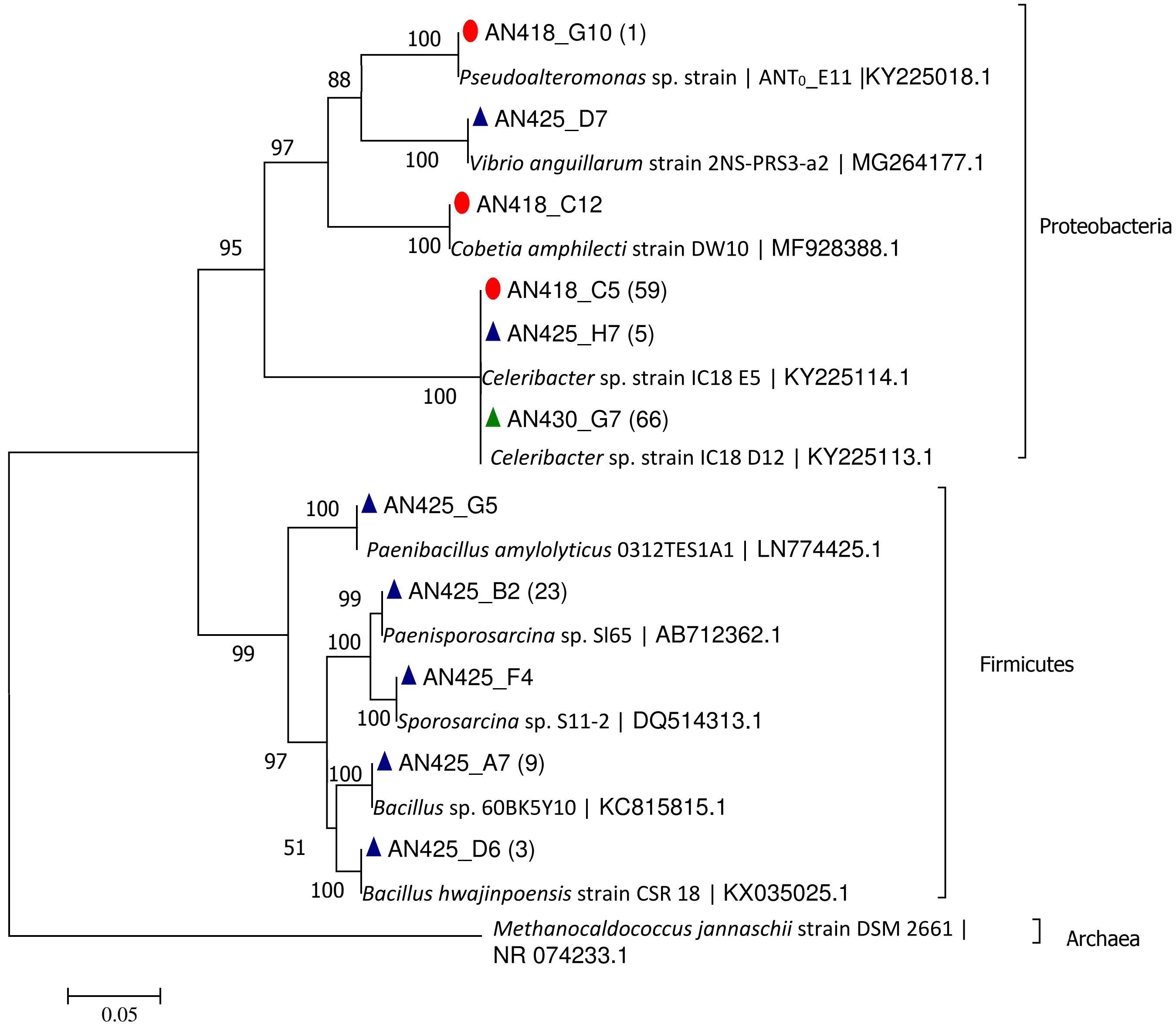

Figure S5

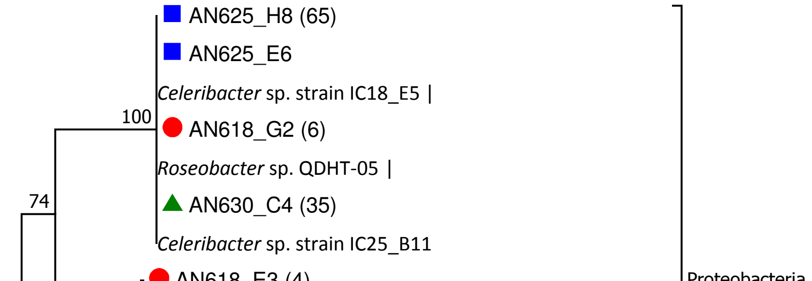

marine drugs

MDPI

Figure S6

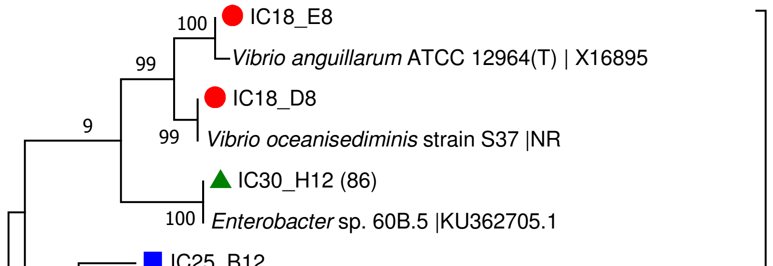

marine drugs

MDPI
